# The Notijoves Project: Protocol for a Randomized Controlled Trial About New Communication Technologies and Gamification to Promote Partner Notification of Sexually Transmitted Infections Among Young People

**DOI:** 10.2196/12896

**Published:** 2019-05-26

**Authors:** Dolors Carnicer-Pont, Eva Loureiro-Varela, Josep Mª Manresa, Montse Martinez, Àngels Avecilla, Laura Montero-Pons, Gemma Falguera-Puig

**Affiliations:** 1 Cancer Prevention and Control Programme Catalan Institute of Oncology Hospital Duran i Reynals L'Hospitalet de Llobregat Spain; 2 Informatics Department Catalan Institute of Oncology Hospital Duran i Reynals L'Hospitalet de Llobregat Spain; 3 Research Suport Unit Metropolitana Nord Primary Health Research Institute Barcelona, Spain Spain; 4 Nursing Department Authonomous University of Barcelona Cerdanyola del Vallès Spain; 5 ASSIR Cerdanyola del Vallès Metropolitana Nord Institut Català de la Salut Barcelona Spain; 6 Badalona Assistential Services Badalona Spain; 7 ASSIR Santa Coloma de Gramanet Metropolitana Nord Institut Català de la Salut Barcelona Spain; 8 ASSIR Sabadell, Metropolitana Metropolitana Nord Institut Català de la Salut Barcelona, Spain Spain

**Keywords:** mobile apps, sexually transmitted infections, partner notification, youth, games, experimental, game theory, randomized controlled trial

## Abstract

**Background:**

An increase in sexually transmitted infections (STIs) as well as an increase in the use of new information and communication technologies among young people in Catalonia is the inspiration behind the idea of designing a smartphone app to promote partner notification of STIs.

**Objective:**

The main objective of this study is to design a Web-based tool adapted to smartphones for partner notification of STIs among youth who are 16 to 24 years old. Additionally, the objective is to evaluate the Web-based tool’s role in increasing the *patient referral* partner notification.

**Methods:**

This is a multicenter randomized controlled trial with a proportional stratification of the sample by center and random allocation of participants to the 3 arms of the study (simple Web-based intervention, game Web-based intervention, and control). This study is being conducted by midwives, gynecologists, and physicians in the sexual and reproductive areas of the primary health care centers.

**Results:**

The primary outcome measure is the number and proportion of partner notifications. Additional outcome measures are the yield of early diagnosis and treatment of those exposed and infected, acceptability, barriers, and preferences for partner notification.

Expected results include an increase in the yield of partner notification, early diagnosis and treatment among youth using Web-based interventions compared with those receiving the traditional advice to notify, and a description of sexual networks among those participating in the study.

**Conclusions:**

The *Notijoves* is expected to have a sustainable positive impact in the partner notification practice among youth and contribute to increasing the awareness of STI prevention.

**International Registered Report Identifier (IRRID):**

DERR1-10.2196/12896

## Introduction

### Background and Study Rationale

Prevalence of sexually transmitted infections (STIs) among young people has increased over the years. In Catalonia, prevalence of *Chlamydia trachomatis* among those below 25 years increased from 5.8% in 2008 to 8.5% in 2013 [1]. The most affected age group is the 16 to 18 years old.

The main rule to control communicable diseases is guaranteeing an early detection of the infected to treat them and alert the exposed [2]. Therefore, sexual partner notification is crucial to reach awareness of the exposed and facilitate testing for potential diagnosis. Scientific evidence highlights the need to improve partner notification and early testing [3-5], and in the case of *Chlamydia trachomatis,* partner notification is proved to be more cost effective than systematic screening [6,7]. Partner notification is recognized to be crucial for reducing transmission and preventing reinfection at an individual level [3].

In our country, as well as in most of the other European countries, the most frequently used models of partner notification are the ones done by the patient itself *(patient referral)*, and the one done by the health professional after obtaining the patient’s approval *(provider referral)* [8].

An internet-based system of partner notification allows the anonymous practice through a Web-based notification, an email, or short messaging system. This tool may help in getting in touch with previous sexual partners with whom there is no interest to revisit in person.

In addition, the introduction of a gamified approach of partner notification among youngsters is an innovative way to involve those who otherwise would not be interested in notifying. By discovering through gamification the main aspects of the STI that the youngsters were diagnosed with and the importance of notifying partners for the prevention of further transmission, the youngsters are more likely to notify their partners about a potential infection, and they are more likely to advise them to get tested for the potential infection.

Provided that youngsters are prone to using new technologies and video games and that previous studies prove the utility of gamification as an important tool to promote behavior changes [9-12], we propose to include this gamification as a third arm of the trial.

This study also includes the social network analysis that allows for a deep study of the relationships among young people. It is based on the evidence that transmission of sexual infections in a social group is determined by the existence of central members within the social structure [13]. Therefore, we aim to conduct a randomized controlled trial to assess the effectiveness of partner notification through the use of new information and communication technologies (ICTs). Our hypothesis is that the percentage of partners informed by their index cases will be higher when the index cases can use new technologies (personally or anonymously) compared with the use of paper notification card to do a *face-to-face* partner notification, without any specific guidance.

### Development of an App to Promote Patient Referral Partner Notification

**Figure 1:**
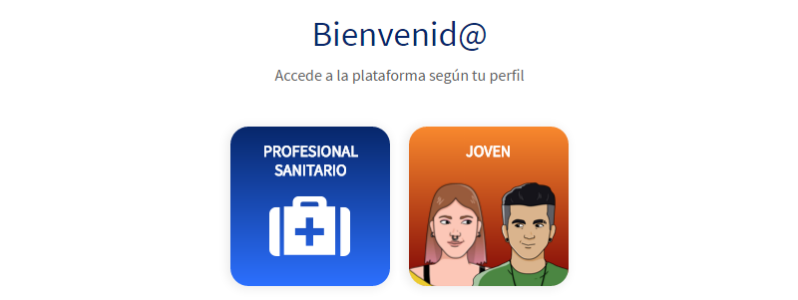
App icon “in Spanish”.

According to the Report of the Spanish Digital Society, 2017, 86% of the youngsters aged 15 to 24 years own a smartphone, and this has become their main means of communication. It is expected that in 2018, 50% of these youngsters will exclusively use smartphones to be on the Web [14]. In addition, these youngsters are heavy users of smartphone apps. Making available a responsive smartphone app that helps to do partner notification, either personally or anonymously, may increase partner notification practice. New and innovative ways of addressing partner notification in a sustainable way that can benefit youngsters and reduce transmission are a high priority. That is why we proposed the development of a smartphone app in Spanish and in Catalan (see Figure 1 for app icon in Spanish). Experts in gamification and Web app contributed to its development, drawing on the gamification-information- motivation-behavioral skills approach to behavioral change [15].

A total of 3 workshops with youngsters of different social levels were conducted by a psychologist, anthropologists, and nurses to identify the best way to design the game.

The app contains different story games that are sensitive to sexual orientation of youngsters, adapted information to the STI that was diagnosed to the patient, and choice of different methods to do partner notification (short message service, email, WhatsApp, or face to face), either anonymously or by identifying himself or herself. At the end of the game, the youngsters will have learned preventive features of the infection, and they should be motivated to inform all sexual partners exposed to the STI; in addition, they will choose a method to send them the message or inform them personally.

### Aims and Objectives

The aim of this study is to describe the protocol for the randomized controlled trial of the *Notijoves* Web-based tool, adapted to smartphones for partner notification of STIs among youngsters aged 16 to 24 years.

The first objective of the study is to design the tool and evaluate its role in increasing partner notification done by the patient (patient referral partner notification) for all eligible sexual partners.

The specific objectives are the following:

- Describe sociodemographic characteristics of index cases and consecutive sexual partners
- Describe coinfections within the group
- Describe acceptability, barriers, and preferences for executing partner notification using ICTs
- Evaluate the utility to apply the game theory (gamification to promote behavior change) in the design of a Web app for partner notification of STIs
- Evaluate the yield of ICTs for partner notification of STIs among sexual partners
- Assess the impact of ICTs in primary transmission of STIs
- Describe the characteristics of the social and sexual network among youngsters

## Methods

### Design

This is an unblinded 3-armed randomized controlled trial, assigning patients to each arm of the study through the proportionally stratified sample of patients by health center, which will test the primary hypothesis that a greater proportion of partners will be informed about their exposure to STIs (syphilis, chlamydia, or gonococcus) in the intervention groups compared with the control group. We will randomize 416 young and recently diagnosed index cases in 1 of the 7 centers of the study. The researcher responsible for analyzing the data will be blind to group allocation.

### Ethical and Research Governance Approval

This trial was approved by the Ethics Committee of the Institute of Research in Primary Care of Catalonia Institut Universitari d’Investigació en Atenció Primària Jordi Gol and given funding through the competitive call: Health Research and Innovation Strategic Plan 2016-2020 of the Catalan Government (ID:SLT002/16/00197).

### Participants

This trial is being conducted in 7 public-based primary health care centers for sexual and reproductive health of Catalonia in the cities of Mataró, Badalona, Santa. Coloma de Gramanet, Granollers, Mollet, Cerdanyola-Ripollet, and Sabadell. Participants to the study are being recruited in each of the primary health care centers according to the following *inclusion criteria*: Young people 16 to 24 years old, diagnosed with at least 1 of the curable STIs (syphilis, gonococcus, or chlamydia). Partners who have had sexual contact within the previous 6 months since the diagnosis of index case.

*Exclusion criteria* are the following: diagnosis of other STIs (trichomonas, HIV, Hepatitis C, and Hepatitis B), which, although partner notification is advisable, will not be included to prevent complexity in the study. Under the diagnosis of 1 of these STIs, health professionals will perform according to the current clinical guidelines.

### Study Period

The study will be conducted within a 3-year period. A total of 1 year to prepare the Web app, 18 months for the field work, and 6 months for the evaluation and dissemination of results.

### Groups of the Study

#### Control Group

Those receiving the recommendation to inform their partners about exposure to STIs by the standard procedure currently available in the Catalan guidelines, use of a Partner notification paper card.

#### Game Intervention Group

Those advised to enter the *Notijoves* Web app with a code and follow the steps of the game to finally choose among different ways to notify their partners.

#### Web App Direct Intervention Group

Those advised to enter the *Notijoves* Web app with a code and directly choose among different ways to notify their partners.

### Main Outcome Variable

The main outcome variable of the study is the percentage of partners informed of their exposure to a STI from their index cases, according to the assigned study group.

### Sample Size

Young population accessing all centers of study within a year is around 400. They have been randomly distributed in 3 arms, according to the Bonferroni correction, to allow multiple comparisons of outcomes with a significance level of *P*=.016. The trial is expected to have 10% of withdrawals after recruitment within the intervention groups and 20% of withdrawals after recruitment within the control group. Therefore, to have at least 120 participants in each 1 of the groups, 133 are recruited in each 1 of the intervention groups and 150 are recruited in the control group. The random list ratio is 1:1:1.28. To achieve this sample, participant recruitment is allowed for a period ranging from 13 to 18 months.

### Randomization and Informed Consent Procedure

The sample is proportionally being assigned to each of the 7 centers, according to their regular activity (see Table 1).

The health professional proposes that the young patient index case should participate in the study, and once verbal acceptance is obtained, he gets into the Web app (see Figure 1) to obtain a random code that assigns the patient to 1 of the 3 arms of the study. He also prints the informed consent to be signed by the patient and explains all the procedures to him/her, according to the arm assigned (control, direct Web app, and game Web app). A copy of the Informed consent is given to the patient, and the original is kept under the custody of the health professional in the participating center.

### Participation Procedure

**Figure 2:**
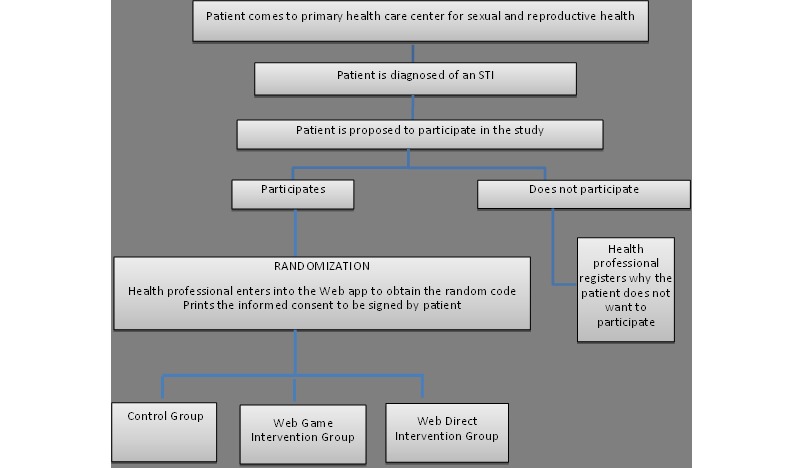
Algorithm of participation.STI: sexually transmitted infection.

All the candidates who accept to participate in the study are given information about the study, both verbally and by receiving an Information sheet. The candidates refusing participation are also asked to complete a quick survey (2 questions) to provide reasons for not participating. (see Figure 2)

### Control Group

The control group receives usual partner notification advice, and the health professional prints as many partner notification paper cards as the patient index case mentions to be able to provide partner notification paper cards to exposed partners. Therefore, exposed partners are expected to receive a partner notification paper card and a letter that are to be given to their health professional. The letter informs of the potential diagnosis and advised treatment. The card has information of the STI and the list of Sexual and Reproductive Health Centers and primary health care centers for sexual and reproductive health, where the recipient of the notification can go for diagnosis and potential treatment.

The random code given to the index case patient appears in the informed consent and in a short paper questionnaire (12 questions). Each 1 of the partner notification paper cards has a code in line with the study arm of the index case. This means that partners receiving a partner notification paper card from an index case assigned to the control arm are kept in the control arm themselves.

**Table 1:** Sample size distribution for each primary health care center for sexual and reproductive health.

Location of health care centres^a^	Control^b^ N1	Intervention game^c^ N2	Intervention Web^d^ N3	Total N
Badalona	30	26	26	82
Sabadell	27	24	24	76
Granollers	27	24	24	74
Mollet	20	18	18	57
Mataró	20	17	17	55
Santa Coloma	14	13	13	40
Cerdanyola	12	11	11	34
Total	150	133	133	416

^a^Primary Health Care Centres for Sexual and Reproductive Health.

^b^Control group.

^c^Game intervention group.

^d^Web app direct intervention group.

### Web App Game Intervention Group

Individuals assigned to this group receive an Information sheet explaining the objectives and procedures of the study. After providing a signature of their informed consent, they receive a paper copy of this informed consent that has a specific code through which they enter the game by connecting to the *Notijoves* Web address. A paper card with the “Notijoves” Web address is given to the patients. Once they enter the Web app, they are asked to feed the given code into the field of Young PANDORA screen.

By entering the code, they start playing the game, and they are directed through different screens and situations. A Web-based questionnaire is inserted in the game. At the end of the game, they are also asked to respond to a satisfaction questionnaire.

The game is expected to motivate the youngster to notify partners of their exposure to an STI. While playing the game, the youngsters are asked to answer questions related to the clinical symptoms, prevention, and control of their recently diagnosed STI. Like this, they *learn by playing.* Another feature of the Web app is the link to advisory videos on how to approach the *face-to-face* notification of exposure to an STI. Finally, the game ends with a screen, where the youngsters can choose from among the different electronic tools to notify (short message service, email, or WhatsApp) either anonymously or by identifying themselves.

The electronic partner notification card has the following: (1) a code automatically assigned by the electronic system that is concordant with the arm where the index case patient entered the study, (2) the informative message advising the partner to visit a health center for diagnosis and potential treatment, and (3) a link to a list of health centers of the study and the Web app address, where the partner could go and where the informed partner could enter and participate in the study by introducing the code. Along with the notification card, the partner receives an electronic letter to be given to the health professional for him to be aware of the STI diagnosed to the sender of this notification and the advised treatment.

### Web App Direct Notification Intervention Group

**Figure 3:**
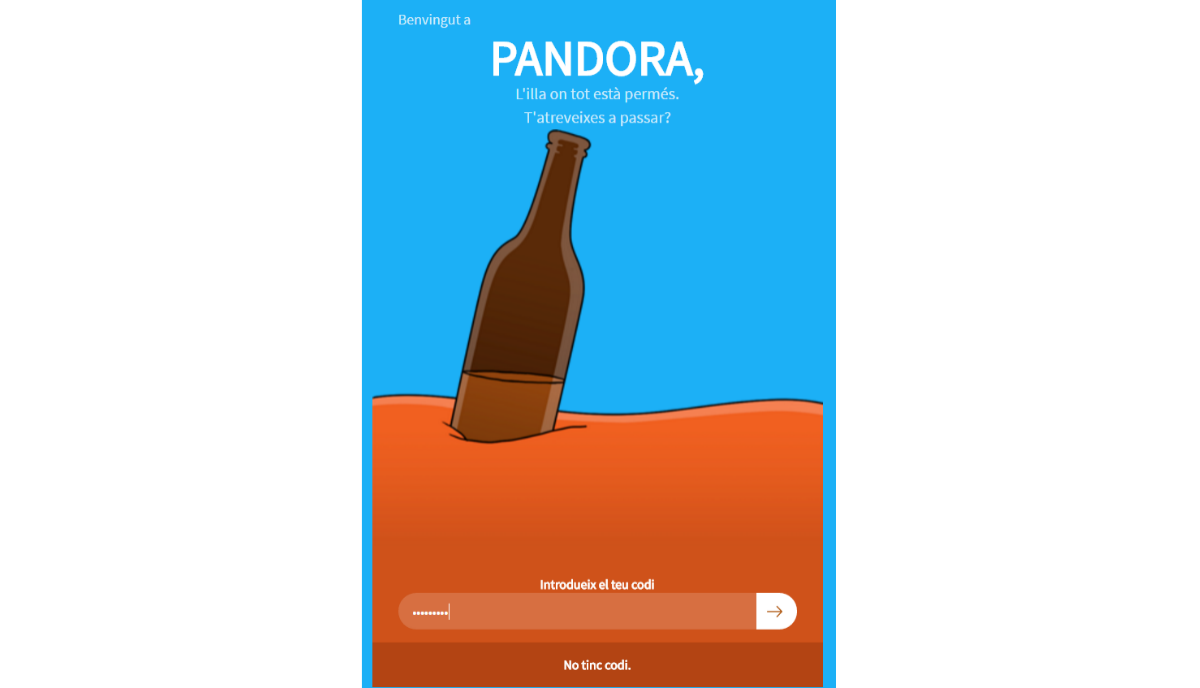
Entrance to game “in Catalan”.

Individuals assigned to this group receive an Information sheet explaining the objectives and procedures of the study. After providing a signature of their informed consent, they receive a paper copy of this informed consent that has a specific code through which they enter the *Notijoves* Web address. A paper card with the *Notijoves* Web address is given to the patients. Once they enter the Web app, they are asked to feed the given code into the field of Young PANDORA screen (see Figure 3). By entering the code, they are directed to a Web-based questionnaire and are offered an option to choose from among the different electronic tools (short message service, email, or WhatsApp) to notify either anonymously or by identifying themselves. Finally, they are also asked to respond to a satisfaction questionnaire.

### What Happens to the Partners Receiving the Notification Card?

Partners visiting participating centers are easily linked to the study by their code, and they are also offered participation to the study. Once accepted, they also sign the informed consent, and they are asked to respond to a recipients’ questionnaire. After receiving the test results, if they are infected, they initiate the same procedure as an index case, with their given code, in concordance with the branch of the study that their index case was assigned. If they are not infected, the health professional registers results linked to the receptors questionnaire.

There may be partners visiting participating centers but not bringing the letter for the health professional and not having the code. These partners cannot be linked to any partner notification card, and therefore they are lost to follow-up for the system. Nevertheless, if they turn out to be infected, they are recruited as new index cases, with a new randomly assigned code. Moreover, those partners visiting a center out of the study area are lost to follow-up for the study.

### Data Collection

The Web app has 2 entries: the entry of health professional and the entry of the youngster.

The health professional entering the Web system activates the random assignment of the code to 1 of the 3 arms of the study and registers the information on the diagnosed STI.

The involvement of the youngsters with the study gives rise to different situations among candidates:

1. The index case wanting to participate
2. The recipient of the notification wanting to participate
3. The index case or the recipient not wanting to participate
4. Participants assigned to 1 of the intervention groups who finalized the experience of partner notification after using the Web app

For each 1 of the situations, there is a specific questionnaire:

1. The questionnaire given to index cases collects data on the recruiting health care center, date of interview, date of birth, sex (boy, girl, transgender born as man, and transgender born as woman), country of birth, mother’s country of birth, education (without studies, primary studies, secondary studies, and university), with whom they have sex (boys, girls, or indistinctly boys or girls), number of sexual partners 6 months before the diagnosis, whether they look at webpages to obtain information about STIs (yes, no), whether they look for partners on the Web (yes, no), currently diagnosed with STI, positioning toward partner notification (will inform all partners, only some, none, and do not know), and reasons for not notifying.
2. The questionnaire given to recipients of a partner notification card collects the same data as above and substitutes the last 3 questions with to which STI have you been exposed? How did you get to know your exposure (face to face, partner notification card, WhatsApp, short message service, email, and other)? Did you receive the notification anonymously (yes, no)?
3. Those not wanting to participate are asked about their reasons in an open question. They are also recorded as index case or recipient of a notification.
4. The opinion or satisfaction questionnaire collects data on whether the Web app helped its users to solve doubts related to STIs (not at all, a few, normal, quite a lot, and a lot), usefulness of the Web app to notify partners (not at all, a few, normal, quite a lot, and a lot), use of the anonymous option to notify (yes, no), easiness to choose from among the options to notify (yes, no, and if not, why not?), wish to recommend the Web app to friends (yes, no, and if not, why not?), and how can we improve the Web app? (open question)

### Data Analysis

Descriptive analysis of index cases visiting health centers will be baselined to further assess differences in yield by groups. Chi-square test and Student *t* test for categorical and continuous variables will be used to assess differences in baseline characteristics among groups. Regression analysis and adjusting for confounding variables will be used to evaluate the primary outcome and relative risk, and its 95% CIs will be calculated. Significance will be set at .05. The multiple imputation method will be used to generate possible values for missing values. This is considered the gold standard for dealing with missing values. Data will be analyzed in Stata v12 (StataCorp LLC).

The methodology of analysis of the social and sexual networks will be used from an egocentric perspective. Structural measures of the network will be calculated, such as the size of the network, number of direct links, density, the geodesic average distance, diameter of ego’s network, and measures of centrality, such as the degree of intermediation of a contact or index case. Furthermore, we will compare social network, density, and structure. Network measurements will be done using the R statistical package.

### Confidentiality, Storage, and Archiving

Data will be archived in a safe environment, according to the European Data protection law.

### Ethical Aspects

The investigators are committed to respect the prevailing norms of Good Clinical Practice, the requisites of the Declaration of Helsinki, and the clauses of general and particular ethical conditions related to the right to privacy, anonymity, and confidentiality. Neither the first name nor surname or any other type of data indicating the identification of the young population will be registered. Therefore, identification will be made by alphanumeric codes. Youngsters participating in the study will sign an Informed Consent by paper, and when they are enrolled through the Web, they will confirm acceptance to participate by clicking on the confirmatory cell.

## Results

The Web app was available to the health professional to initiate recruitment by early April 2018. From April 1 to May 30, we expected to recruit 83 index cases, but difficulties on accreditation of new health professionals and on entry to the study environment in the Web prevented recruitment. In early June 2018, there were 32 young people who accepted to participate in the study. They were assigned randomly to control group (n=15), game intervention group (n=9), and Web direct intervention group (n=8).

The project is currently recruiting with a rate of approximately 6 recruitments per month. Recruitment is slower than expected. Mainly because of the difficulties in the implementation of the Web app. Our confidence is that within the last 6 months of the field study, recruitment pace will be doubled, and the expected sample size will be achieved. To improve recruitment, since September 2018, we have allocated 3 more professionals to the task.

## Discussion

### Summary

During the trial period, the Web app is only available to research participants; therefore, source coding and App content have been preserved.

### Limitations

First, the study relies on an assumed independence of the groups. This means that young participants randomly assigned to 1 of the intervention groups will not share information with those falling in the control group. However, it can happen that some controls have friends assigned to the game or Web app directly and that they visit it with them. This can generate a classification bias, underestimating the real effect of the intervention. Second, information bias to assess primary transmission can occur when not all expected partners to be notified are the ones who visit health centers for testing. Third, those partners receiving a notification but visiting a health center out of the study area will be lost to follow-up if they are assigned to the control group. This will generate a participation bias, overestimating the partner notification yield of the other arms of the study. Fourth, a malicious use of the Web app could occur, when anonymous partner notification is sent by some youngsters to other youngsters who were not even exposed. This may generate overestimation of the partner notification yield. Finally, we cannot fully ascertain the network across experimental conditions because of the anonymous condition of our experiment. It may happen, although very rarely, that partners receive a notification from 2 index cases assigned to different arms of the trial. In that case, the system assumes that the first notification received by the youngster is the one that will be followed-up and the one that will contribute to network analysis.

### Conclusions

Few studies have examined the yield of partner notification among young people, especially by the use of Web apps. The *Notijoves* smartphone app is an intervention that, if shown to be effective, may be implemented for all of Catalonia.

## Abbreviations

ICT: information and communication technology
STI: sexually transmitted infection
